# PriorSAM-DBNet: A SAM-Prior-Enhanced Dual-Branch Network for Efficient Semantic Segmentation of High-Resolution Remote Sensing Images

**DOI:** 10.3390/s26020749

**Published:** 2026-01-22

**Authors:** Qiwei Zhang, Yisong Wang, Ning Li, Quanwen Jiang, Yong He

**Affiliations:** 1 College of Computer Science and Technology, Guizhou University, Guiyang 550025, China; gs.qwzhang23@gzu.edu.cn (Q.Z.); yswang@gzu.edu.cn (Y.W.); gs.lbwu23@gzu.edu.cn (N.L.); gs.lkfeng23@gzu.edu.cn (Q.J.); 2 State Key Laboratory of Public Big Data, Guizhou University, Guiyang 550025, China

**Keywords:** remote sensing, semantic segmentation, Segment Anything Model (SAM), deep learning, sensor data fusion, high-resolution imagery, intelligent perception

## Abstract

Semantic segmentation of high-resolution remote sensing imagery is a critical technology for the intelligent interpretation of sensor data, supporting automated environmental monitoring and urban sensing systems. However, processing data from dense urban scenarios remains challenging due to sensor signal occlusions (e.g., shadows) and the complexity of parsing multi-scale targets from optical sensors. Existing approaches often exhibit a trade-off between the accuracy of global semantic modeling and the precision of complex boundary recognition. While the Segment Anything Model (SAM) offers powerful zero-shot structural priors, its direct application to remote sensing is hindered by domain gaps and the lack of inherent semantic categorization. To address these limitations, we propose a dual-branch cooperative network, PriorSAM-DBNet. The main branch employs a Densely Connected Swin (DC-Swin) Transformer to capture cross-scale global features via a hierarchical shifted window attention mechanism. The auxiliary branch leverages SAM’s zero-shot capability to exploit structural universality, generating object-boundary masks as robust signal priors while bypassing semantic domain shifts. Crucially, we introduce a parameter-efficient Scaled Subsampling Projection (SSP) module that employs a weight-sharing mechanism to align cross-modal features, freezing the massive SAM backbone to ensure computational viability for practical sensor applications. Furthermore, a novel Attentive Cross-Modal Fusion (ACMF) module is designed to dynamically resolve semantic ambiguities by calibrating the global context with local structural priors. Extensive experiments on the ISPRS Vaihingen, Potsdam, and LoveDA-Urban datasets demonstrate that PriorSAM-DBNet outperforms state-of-the-art approaches. By fine-tuning only 0.91 million parameters in the auxiliary branch, our method achieves mIoU scores of 82.50%, 85.59%, and 53.36%, respectively. The proposed framework offers a scalable, high-precision solution for remote sensing semantic segmentation, particularly effective for disaster emergency response where rapid feature recognition from sensor streams is paramount.

## 1. Introduction

High-resolution remote sensing image semantic segmentation is a cornerstone technique for the automated processing of complex earth surface scenes, facilitating applications from smart city sensing to disaster monitoring. With the rapid advancement of satellite and drone sensor technologies, the volume of remote sensing data has expanded explosively [1]. However, the complexity of dense urban scenarios—characterized by the coexistence of multi-scale objects, severe signal occlusion (e.g., shadows from high-rise buildings), and intricate boundaries—poses significant challenges for automated interpretation algorithms. Existing methods often struggle to simultaneously model fine-grained local details and global contextual semantics from the acquired sensor data.

Mainstream single-branch architectures face inherent structural bottlenecks. Convolutional Neural Networks (CNNs) [2,3,4,5,6], while effective at local feature extraction, suffer from limited receptive fields, often leading to the omission of small targets or discontinuity in large-scale structures like roads. To alleviate these issues, recent lightweight CNN architectures like SC-SKNet [7] have been proposed for real-time segmentation, yet the trade-off between efficiency and large-scale context modeling remains unresolved. Conversely, Vision Transformers (ViTs) [8,9] excel at capturing global dependencies but face quadratic computational complexity with respect to image resolution [10], and their patch-based processing can blur high-frequency boundary information during downsampling operations.

To address the computational burden of Transformers, seminal works from the past few years have explored hierarchical structures. Notably, SegFormer [11] introduced a positional-encoding-free design with a lightweight MLP decoder, establishing a strong baseline for efficient semantic segmentation. Similarly, Xu et al. proposed the Efficient Transformer [12] to specifically tackle edge classification issues in remote sensing imagery while reducing inference costs. Despite these advancements, balancing the “efficiency–accuracy” trade-off in dense urban scenarios with limited training data remains a persistent challenge.

Recent hybrid approaches have attempted to mitigate this. The Densely Connected Swin (DC-Swin) Transformer [13] combines shifted window attention with dense feature aggregation. Furthermore, advanced variants like FML-Swin [14] and Swin-MDFF [15] have introduced feature interactive fusion and multi-scale dilated mechanisms to enhance small object detection in urban scenes. Despite these improvements, training such complex models from scratch on limited remote sensing datasets can lead to overfitting or poor generalization on unseen architectural styles.

Recently, the Segment Anything Model (SAM) [16] has shown potential as a foundation model for visual tasks. However, directly applying SAM to remote sensing is problematic due to the significant domain gap between natural and satellite imagery [17,18]. SAM generates class-agnostic masks, which lack the specific semantic category information required for tasks like land-cover classification. Recent foundation model adaptations, such as RSAM-Seg [19] and SAM2Former [20], have attempted to bridge this gap by integrating adapter modules. However, the massive parameter size of SAM makes full fine-tuning computationally prohibitive for many applications [21].

To address these efficiency and generalization challenges, recent studies have proposed innovative adaptation frameworks. Notably, SpectralX [22] introduces a parameter-efficient fine-tuning strategy that leverages attribute-refined adapters to achieve robust domain generalization for spectral remote sensing foundation models without extensive retraining.

To bridge this gap, recent works have explored various fusion strategies. Some methods employ full-parameter fine-tuning, which demands extensive computational resources. Others use prompt engineering (e.g., RSPrompter [23]), which can be brittle and labor-intensive to design for diverse scenes. Approaches like MC-SAM SEG [24] and CWSAM [25] utilize parameter-efficient adapters to transfer SAM to optical and SAR domains, respectively. However, these approaches often fail to strike an optimal balance between efficiency (parameter count/inference speed) and accuracy (boundary precision/semantic correctness).

To address these limitations, we propose PriorSAM-DBNet, a dual-branch collaborative framework designed for efficient sensor data interpretation. Unlike previous methods that view SAM merely as a feature extractor, we conceptualize the frozen SAM backbone as a “Structural Prior Generator” that provides a rigid boundary constraint to the learnable “Semantic Encoder” (DC-Swin Transformer). Our approach introduces a novel adaptation strategy via the Scaled Subsampling Projection (SSP) module, which utilizes shared weights across scales to align features efficiently. This design aligns with the emerging trend of dual-branch architectures in remote sensing, such as TDBAN [26] and DBSANet [27], but distinguishes itself by explicitly leveraging zero-shot priors to enhance signal interpretation. Our core innovation lies in the synergistic integration of these two branches via the proposed Attentive Cross-Modal Fusion (ACMF) module. Rather than performing simple feature concatenation, the ACMF module leverages high-fidelity spatial priors from SAM to calibratethe semantic features from the Swin Transformer, effectively resolving semantic ambiguities in boundary regions.

The main contributions of this study are as follows:

(1) A dual-branch collaborative architecture is proposed that synergizes the global contextual modeling of the DC-Swin Transformer with the robust object-boundary priors of SAM. This design effectively resolves the trade-off between semantic consistency and boundary precision in dense urban scenes.
(2) A lightweight adaptation strategy is introduced, comprising a frozen SAM backbone and a novel Scaled Subsampling Projection (SSP) module. By enforcing a shared-weight projection logic across scales, this strategy achieves multi-scale feature alignment with minimal trainable parameters (0.91 million), ensuring high efficiency suitable for real-time sensor data processing applications, comparable to recent lightweight models like LightFormer [28].
(3) The Attentive Cross-Modal Fusion (ACMF) module is developed as a dynamic mechanism that enables adaptive interaction between the prior and semantic branches. By explicitly modeling the correlation between structural integrity and semantic class, ACMF significantly improves segmentation consistency in complex boundary regions.

## 2. Related Work

### 2.1. Semantic Segmentation in Remote Sensing

The evolution of semantic segmentation in remote sensing has transitioned from handcrafted feature engineering to deep learning paradigms characterized by increasingly sophisticated context modeling. Early dominance was established by Convolutional Neural Networks (CNNs), particularly Fully Convolutional Networks (FCNs) [3] and symmetric encoder–decoder architectures like U-Net [4]. While effective in local feature extraction, standard CNNs suffer from limited receptive fields, leading to inadequate modeling of long-range dependencies essential for large-scale geospatial objects [2]. To mitigate this, mechanisms such as atrous spatial pyramid pooling (ASPP) [5] and pyramid pooling modules [6] were introduced to aggregate multi-scale context. However, these operations often incur a loss of high-frequency spatial details, resulting in coarse boundaries for small, dense targets in urban scenarios.

The advent of Vision Transformers (ViTs) [8] marked a paradigm shift towards global context modeling, as detailed in recent comprehensive surveys [29]. In the remote sensing domain, architectures like Swin-Unet and TransUNet [30] have leveraged self-attention mechanisms to capture long-range semantic dependencies. To combine the strengths of both paradigms, hybrid architectures have emerged. For instance, Swin Transformer Embedding UNet (ST-UNet) [31] proposed a dual-encoder structure combining CNNs and Transformers to enhance feature representation. Similarly, UNetFormer [32] utilized a lightweight ResNet encoder with a Global–Local Transformer Block (GLTB) decoder for efficient urban scene segmentation. These works demonstrate that dual-branch or hybrid designs can effectively synergize local texture extraction with global semantic modeling.

Specifically, the DC-Swin Transformer [13] combines hierarchical shifted window attention [9] with densely connected feature aggregation, effectively balancing representation power and computational efficiency. Recent advancements, such as LSENet [33] and AFNE-Net [34], have further augmented these architectures with spatial enhancement modules to refine local details.

Despite these strides, training pure Transformer models from scratch on limited remote sensing datasets poses significant risks of overfitting and typically requires massive annotated data to converge [14]. Furthermore, pure semantic encoders often struggle to decouple distinct objects in dense clusters due to boundary blurring during patch embedding. This has catalyzed interest in adapting foundation models, such as the Segment Anything Model (SAM) [16], which offers robust, zero-shot structural priors. However, the direct application of SAM is hindered by the domain gap between natural and satellite imagery [17,18]. Recent works like RSPrompter [23] and SAM-Adapter [35] have attempted to bridge this gap, yet effectively integrating SAM’s frozen structural knowledge with learnable semantic features remains an open challenge. PriorSAM-DBNet addresses this by treating SAM not merely as a feature extractor, but as a rigid structural constraint provider within a dual-branch system.

### 2.2. Foundation Models and SAM Adaptation

The Segment Anything Model (SAM) [16] has emerged as a robust foundation model for image segmentation. Its zero-shot capabilities have been explored in various domains, including medical imaging [36] and camouflage detection [35]. In remote sensing, the application of SAM faces challenges due to domain shifts. Recent works like RSPrompter [23] utilize external detection boxes as prompts, while others like SAM-Adapter [35] employ lightweight adapters to tune SAM for specific downstream tasks. The adaptation of SAM for remote sensing has become a vibrant research area in 2024–2025. FastSAM [37] focuses on efficiency and scale awareness. PointSAM [38] investigates point-based supervision for fine-tuning, while recent works like SAM2-UNet [39] and SAM2Former [20] have begun integrating the next-generation SAM 2 encoder into U-shaped architectures. Similarly, Zhang et al. proposed SpectralX [22], a parameter-efficient framework that adapts existing RSFMs using a two-stage training approach. By utilizing an attribute-oriented mixture of adapters, it effectively bridges the gap between pre-trained models and diverse spectral modalities. However, few works have successfully integrated SAM as a structural prior within a dual-branch architecture that preserves the efficiency of a frozen backbone while leveraging the semantic learning power of a separate encoder, as proposed in our PriorSAM-DBNet. This approach shares conceptual similarities with the recent CrossEarth [40] foundation model but focuses specifically on the efficient fusion of priors rather than large-scale pre-training.

## 3. Methodology

### 3.1. PriorSAM-DBNet Architecture Overview

**Figure 1:**
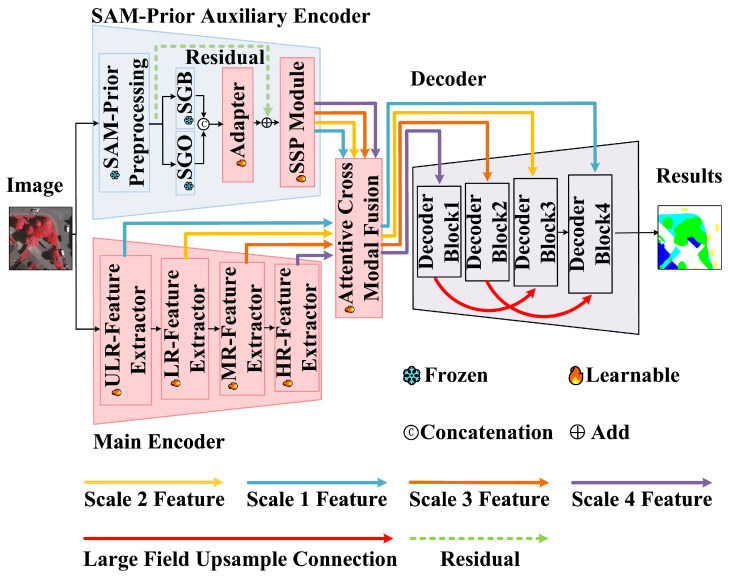
Dual-branch architecture of PriorSAM-DBNet: The SAM-prior auxiliary encoder (top branch) generates object-boundary masks through a frozen ViT-H backbone, serving as a structural guide. The DC-Swin main encoder (bottom branch) extracts hierarchical features via shifted window attention to capture global semantics. The ACMF module performs dynamic cross-modal fusion at four scales, followed by a hierarchical decoder with DCFAM for high-resolution map reconstruction.

As illustrated in Figure 1, PriorSAM-DBNet adopts a parallel dual-branch encoding architecture to exploit the complementary strengths of specific semantic learning and general structural priors. The Main Encoder employs a four-stage DC-Swin Transformer [13] to extract hierarchical semantic features ${\mathrm{ST}}_{1},{\mathrm{ST}}_{2},{\mathrm{ST}}_{3},{\mathrm{ST}}_{4}$. This branch is fully learnable and responsible for capturing the spectral–semantic relationships specific to the remote sensing dataset. The SAM-Prior Auxiliary Encoder utilizes a frozen SAM ViT-H backbone to process image prompts—specifically SAM-Generated Object Masks (SGO) and SAM-Generated Boundary Masks (SGB)—transforming them into hierarchical prior features (${\mathrm{SP}}_{1},{\mathrm{SP}}_{2},{\mathrm{SP}}_{3},{\mathrm{SP}}_{4}$) via the SSP module. These features are fused by the ACMF module and reconstructed by a hierarchical decoder. To rigorously define the data flow, we denote scalars in normal italic (e.g., *k*) and vectors/tensors in bold italic (e.g., F).

### 3.2. Main Encoder and SAM-Prior Auxiliary Encoder

**Figure 2:**
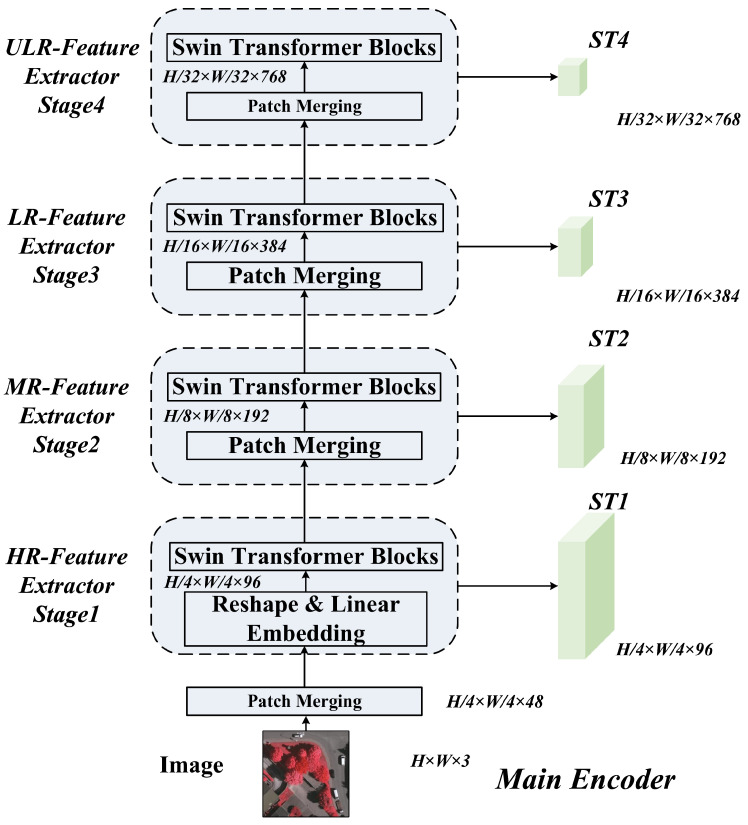
Detailed architecture of the Main Encoder employing the DC-Swin Transformer with four hierarchical feature processing stages.

The Main Encoder follows the DC-Swin Transformer design [13], utilizing Shifted Window Attention [9] to capture long-range dependencies while maintaining linear computational complexity. It generates hierarchical features ${\mathrm{ST}}_{k}$ at four stages. Specifically, the feature map at stage *k* is defined as ${\mathrm{ST}}_{k}\in R^{H_{k}\times W_{k}\times C_{k}}$, where $H_{k}=\frac{H}{2^{k+1}}$, $W_{k}=\frac{W}{2^{k+1}}$, and $C_{k}$ is the channel dimension. The detailed architecture of the Main Encoder is shown in Figure 2.

**Figure 3:**
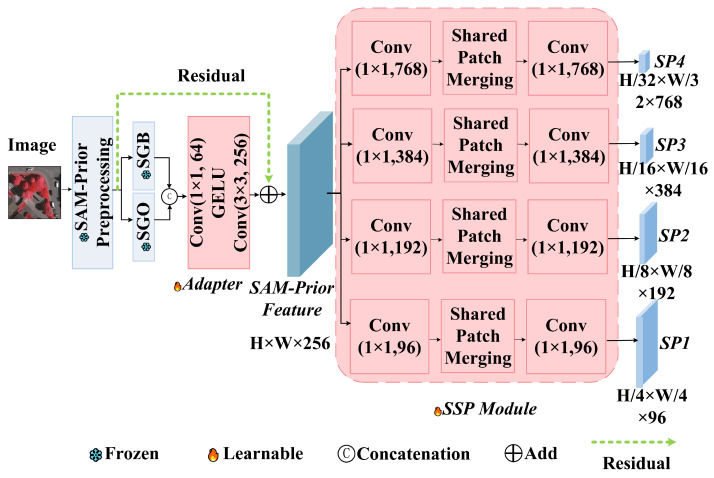
Detailed structure of the SAM-Prior auxiliary encoder. It comprises an Adapter module for initial feature transformation and a Scaled Subsampling Projection (SSP) module. The SSP implements multi-scale resolution alignment using shared convolutional weights across different stages to ensure parameter efficiency and robust feature abstraction.

The Auxiliary Encoder is designed for parameter efficiency. We generate SGO and SGB using a grid-prompted SAM. To ensure the reliability of these priors, we apply a triple filtering strategy: a stability score threshold (0.96) to remove low-confidence predictions, an object quantity limit (K=50) to prevent over-segmentation in textured areas (e.g., tree canopies), and a minimum size threshold (S=50 pixels) to exclude noise. These hyperparameters were empirically verified through sensitivity analysis on the ISPRS Vaihingen dataset, where 0.96 yielded the optimal balance between boundary stability and recall, K=50 reached the efficiency saturation point, and S=50 effectively minimized granular noise while preserving small targets. As shown in Figure 3, these binary masks are first processed by a lightweight adapter. This adapter consists of sequential 1×1 and 3×3 convolutions that project the sparse mask information into a dense 256-channel feature space. The Scaled Subsampling Projection (SSP) module then aligns these features to the resolutions of the main branch (H/4 to H/32) using shared convolutional weights. This weight-sharing mechanism reduces the parameter count of the adapter to just 0.91 million (representing only 0.15% of SAM’s total parameters), preventing the auxiliary branch from overfitting to the limited training data while preserving the generalization power of SAM.

*Theoretical Justification for Shared Weights:* A distinct characteristic of our SSP design is the employment of *shared convolutional weights* across all hierarchical scales. This design choice is grounded in two critical theoretical insights:

- *Enforcing Scale Invariance:* In remote sensing imagery, the fundamental geometric properties of objects (e.g., boundaries and corners) remain topologically consistent across scales. By constraining the projection layers to share weights $\theta_{shared}$ across scales k∈{1,…,4}, we force the network to learn a generalized, scale-agnostic mapping function $f_{\theta }:F_{SAM}\to F_{Semantic}$. This prevents the model from overfitting to scale-specific artifacts and promotes the learning of intrinsic structural representations.
- *Parameter Efficiency and Regularization:* Given the limited size of remote sensing datasets, increasing model complexity risks catastrophic overfitting. Independent adapters for each scale would quadruple the parameter count. By sharing weights, we constrain the auxiliary branch to a minimal 0.91 million trainable parameters (approx. 0.15% of the SAM backbone). This acts as a structural regularizer, ensuring that the limited gradients are concentrated on optimizing a robust, unified projection logic rather than dispersing over redundant, scale-specific parameters.

Mathematically, the projection at scale *k* is defined as

(1) $${\mathrm{SP}}_{k}=D_{↓k}\left(f_{conv}^{1\times 1}\left(F_{prior};\theta_{shared}\right)\right)$$

where $D_{↓k}$ represents the scale-specific downsampling operation and $\theta_{shared}$ denotes the reusable projection kernel. Here, $F_{prior}\in R^{H\times W\times C_{in}}$ represents the initial adapter features, and the output ${\mathrm{SP}}_{k}\in R^{H_{k}\times W_{k}\times C_{out}}$ denotes the aligned structural prior feature tensor matching the resolution of the main branch.

### 3.3. Attentive Cross-Modal Fusion (ACMF) Module

To effectively integrate the semantic features from the main branch with the structural priors from the auxiliary branch, we propose the ACMF module. Simple addition or concatenation is insufficient because the class-agnostic boundaries from SAM may not strictly align with the target semantic classes in complex transition zones. ACMF employs a spatial attention mechanism to dynamically weight the importance of features based on their cross-modal correlation. The explicit algorithmic flow of the ACMF forward propagation is detailed in Algorithm 1. This process ensures that the structural priors are selectively fused only where they reinforce the semantic consistency, effectively suppressing noise.
**Algorithm 1** Forward Propagation of PriorSAM-DBNet with ACMF**Require:** Input Image *I*, Pre-computed SAM Masks $M_{obj},M_{bdy}$**Ensure:** Segmentation Map $S_{map}$*Phase 1: Prior Encoding (Frozen SAM Branch)*Generate initial prior features: $F_{prior}$$\leftarrow \mathrm{Adapter}(M_{obj},M_{bdy})$**for** k=1 to 4 **do**   ${\mathrm{SP}}_{k}$$\leftarrow \text{SSP\_Layer}(F_{prior},\mathrm{scale}=k)$ {Downsampling via Shagreen Weights $\theta_{shagreen}$}**end for***Phase 2: Semantic Encoding (Learnable Main Branch)***for** k=1 to 4 **do**   ${\mathrm{ST}}_{k}$$\leftarrow {\mathrm{SwinBlock}}_{k}\left({\mathrm{ST}}_{k-1}\right)$ {Shifted Window Attention Extraction}**end for***Phase 3: Attentive Cross-Modal Fusion (ACMF)***for** k=1 to 4 **do**   ${\tilde{\mathrm{ST}}}_{k}$$\leftarrow {\mathrm{Conv}}_{1\times 1}\left({\mathrm{ST}}_{k}\right)$ {Channel Alignment}   ${\tilde{\mathrm{SP}}}_{k}$$\leftarrow {\mathrm{Conv}}_{1\times 1}\left({\mathrm{SP}}_{k}\right)$   $U_{k}$$\leftarrow \mathrm{Concat}({\tilde{\mathrm{ST}}}_{k},{\tilde{\mathrm{SP}}}_{k})$ {Feature Aggregation}   $A_{k}$$\leftarrow \sigma ({\mathrm{Conv}}_{spatial}\left(U_{k}\right))$ {Generate Spatial Attention Map}   $F_{k}$$\leftarrow {\tilde{\mathrm{ST}}}_{k}+\beta \;\cdot \;\left(A_{k}⊙{\tilde{\mathrm{SP}}}_{k}\right)$ {Residual Fusion with Learnable Scalar β}**end for***Phase 4: Decoding*$S_{map}\leftarrow \mathrm{Decoder}\left(F_{1},F_{2},F_{3},F_{4}\right)$ {Multi-scale Reconstruction via DCFAM}**return** $S_{map}$

Mathematically, the fusion process at scale *k* is formalized as follows. First, channel alignment is performed:

(2) $${\tilde{\mathrm{ST}}}_{k}=f_{\mathrm{conv}}^{1\times 1}\left({\mathrm{ST}}_{k}\right),\;{\tilde{\mathrm{SP}}}_{k}=f_{\mathrm{conv}}^{1\times 1}\left({\mathrm{SP}}_{k}\right)$$

where $f_{\mathrm{conv}}^{1\times 1}$ denotes the convolutional kernel. The aligned features ${\tilde{\mathrm{ST}}}_{k}$ and ${\tilde{\mathrm{SP}}}_{k}$ share the same dimensions $R^{H_{k}\times W_{k}\times C_{mid}}$.

**Figure 4:**
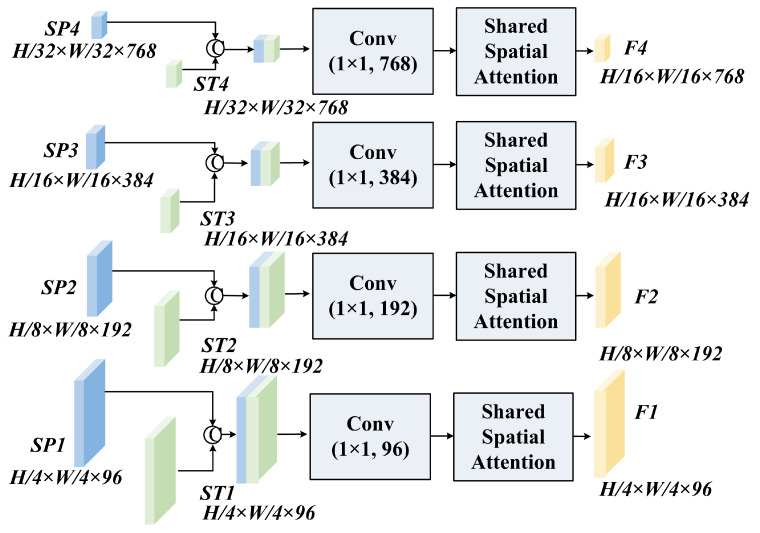
Architecture of the Attentive Cross-Modal Fusion (ACMF) module. This module dynamically fuses features from the main and auxiliary branches using a shared spatial attention mechanism to eliminate semantic ambiguity and align boundaries.

The spatial attention map $A_{k}\in R^{H_{k}\times W_{k}\times 1}$ is computed to highlight regions where the structural prior effectively supports the semantic prediction:

(3) $$A_{k}=\sigma \left(F_{spatial}\left(\right)\right)$$

where σ is the Sigmoid activation function and [;] denotes concatenation along the channel axis. Finally, the fused feature $F_{k}$ is obtained via a residual connection to preserve gradient flow:

(4) $$F_{k}={\tilde{\mathrm{ST}}}_{k}+{\tilde{\mathrm{ST}}}_{k}⊙A_{k}+\beta \;\cdot \;\left({\tilde{\mathrm{SP}}}_{k}⊙A_{k}\right)$$

where β is a learnable scalar parameter and ⊙ represents element-wise multiplication. The resulting fused feature map $F_{k}$ retains the shape $R^{H_{k}\times W_{k}\times C_{mid}}$. This mechanism effectively filters out noise in the semantic branch caused by complex textures by leveraging the high-confidence boundary priors (see Figure 4).

### 3.4. Decoder

**Figure 5:**
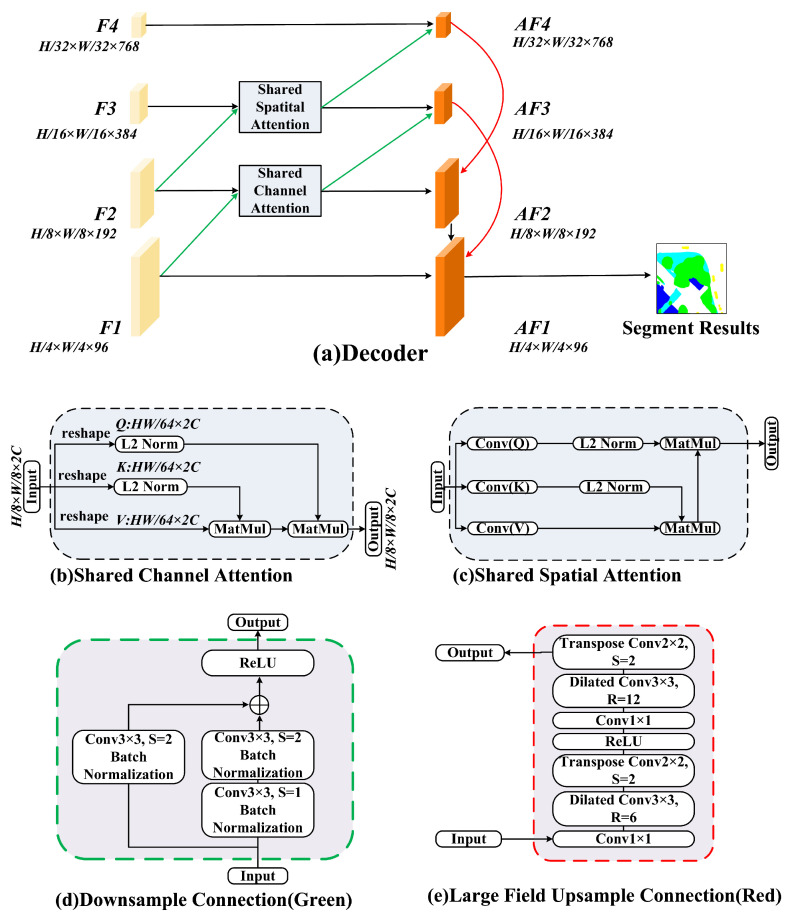
Comprehensive architecture of the Decoder module. (**a**): Illustrates the hierarchical decoding process, utilizing a multi-scale reconstruction strategy to recover high-resolution semantic maps (AF1) from coarse feature representations (F4). (**b**–**e**): Detailed internal structures of the attention and connection mechanisms are shown in the surrounding insets.

The decoder reconstructs high-resolution segmentation maps through dilated receptive field upsampling and shared dual-attention mechanisms, as shown in Figure 5. For hierarchical features at level *k* (k∈{4,3,2,1}), the decoding procedure is formalized as follows.

*Upsampling & Feature Fusion:*

(5) $${\hat{F}}_{k}=U_{2\times 2}\left(D_{r_{k}}\left(F_{k}\right)\right)$$

where $D_{r_{k}}$ denotes a 3×3 dilated convolution with rate $r_{k}$ ($r_{k}=6/12$ for Block4, $r_{k}=2$ for Blocks3-1) and $U_{2\times 2}$ represents transposed convolution upsampling. The upsampled features ${\hat{F}}_{k}\in R^{H_{k-1}\times W_{k-1}\times C_{k-1}}$ are then fused with lower-level features.

*Shared Spatial Attention (SSA):*

(6) $$A_{s}=\sigma \left(f_{\mathrm{conv}}^{3\times 3}\left(\left[{\hat{F}}_{k};F_{k-1}\right]\right)\right)$$

where $A_{s}\in R^{H_{k-1}\times W_{k-1}\times 1}$ is the spatial attention map.

*Shared Channel Attention (SCA):*

(7) $$A_{c}=\sigma \left(f_{\mathrm{conv}}^{1\times 1}\left(\mathrm{GAP}(\left[{\hat{F}}_{k};F_{k-1}\right])\right)\right)$$

where GAP(·) denotes Global Average Pooling, resulting in the channel attention vector $A_{c}\in R^{1\times 1\times 2C_{k-1}}$.

*Feature Aggregation:*

(8) $${\mathrm{AF}}_{k-1}=f_{\mathrm{conv}}^{3\times 3}\left(A_{s}⊙A_{c}⊙\left({\hat{F}}_{k}+F_{k-1}\right)\right)$$

where ${\mathrm{AF}}_{k-1}\in R^{H_{k-1}\times W_{k-1}\times C_{out}}$ serves as the input for the subsequent decoding stage and ⊙ denotes element-wise multiplication. The final Block1 output ${\mathrm{AF}}_{1}$ is refined through 3×3 convolution and 4× bilinear upsampling. Cross-level skip connections dynamically integrate high-level semantics with low-level details, reducing small target omission while maintaining building boundary topology.

### 3.5. Loss Function

We utilize a composite loss function to supervise training, ensuring both pixel-wise semantic correctness and boundary fidelity:

(9) $$L_{total}=L_{CE}+\lambda_{1}L_{obj}+\lambda_{2}L_{bdy}$$

Here, $L_{CE}$ is the standard Cross-Entropy loss for pixel-wise classification. $L_{obj}$ and $L_{bdy}$ are auxiliary Binary Cross-Entropy (BCE) losses derived from the SAM priors to enforce object coherence and boundary sharpness, respectively. Following the sensitivity analysis in [1], we empirically set the hyperparameters $\lambda_{1}=1.0$ and $\lambda_{2}=0.1$ to balance the gradient contributions of each term.

#### Definition of Boundary Metrics

To rigorously evaluate the boundary performance improvements mentioned in the introduction, we explicitly define the *Boundary F1-score (BF1)* [2]. Standard mIoU metrics are often dominated by the large interior regions of objects, masking errors at the boundaries. Let *G* be the set of pixels constituting the ground truth boundaries and *P* be the set of pixels in the predicted boundaries. We define a tolerance buffer distance *d* (set to 3 pixels for our experiments). Precision ($P_{B}$) is defined as the fraction of pixels in *P* that are within distance *d* of a pixel in *G*. Recall ($R_{B}$) is defined as the fraction of pixels in *G* that are within distance *d* of a pixel in *P*. The Boundary F1-score is then calculated as

(10) $$BF1=\frac{2\;\cdot \;P_{B}\;\cdot \;R_{B}}{P_{B}+R_{B}}$$

This metric specifically quantifies the model’s ability to delineate sharp transitions between urban land-cover classes.

## 4. Experiments and Discussion

### 4.1. Datasets and Experimental Setup

We evaluate our method on three benchmark datasets representing diverse urban landscapes:

1. *ISPRS Vaihingen:* Contains 33 patches of size roughly 2500×2000 pixels with a 9 cm resolution. It features 3 bands (IR, R, and G) and depicts a historic German city with dense complex buildings.
2. *ISPRS Potsdam:* Contains 38 patches of size 6000×6000 pixels with a 5 cm resolution. It features 4 bands (IR, R, G, and B) and also depicts a historic city but with different architectural styles.
3. *LoveDA Urban:* A challenging dataset with 30 cm resolution, featuring diverse scenes from urban-to-rural transitions, which tests the model’s generalization across scales.

#### Baseline Selection Criteria

To ensure the rigor and transparency of our comparative analysis, we established specific inclusion criteria for the baseline methods, partially adopting the principles of the PRISMA guidelines [41] for transparent reporting:

- *Relevance:* Selected methods must be specifically designed for or adapted to high-resolution remote sensing semantic segmentation in dense urban scenarios.
- *Recency and Impact:* We prioritized state-of-the-art models published in high-impact venues (e.g., IEEE TGRS, ISPRS P&RS, and CVPR) within the last five years (2020–2025).
- *Architectural Diversity:* To provide a comprehensive evaluation, we included representatives from three distinct architectural paradigms:
  1. CNN-based methods: ABCNet [42] and ShelfNet [43];
  2. Transformer-based methods: DC-Swin [13], UNetFormer [32], and RSMamba [44];
  3. Foundation Model adaptations: MeSAM [45] and SAM-DBnet [46].

This selection strategy ensures that PriorSAM-DBNet is benchmarked against the most competitive and relevant approaches in the field.

Implementation was performed in PyTorch (v2.9.1) on NVIDIA A100 GPUs (Santa Clara, CA, USA). The model is trained with stochastic gradient descent (SGD) with a learning rate of 0.01 and batch size of 16. To ensure a fair comparison, all baseline models and the proposed method were trained using an identical protocol. We employed an adaptive Early Stopping strategy rather than a fixed number of epochs. Specifically, the maximum training duration was set to 200 epochs, with a patience threshold of 15 epochs; training was terminated if the validation loss did not improve for 15 consecutive epochs, and the checkpoint with the lowest validation loss was retained for evaluation.

### 4.2. Performance Comparison

#### 4.2.1. Performance Analysis on the ISPRS Vaihingen Dataset

PriorSAM-DBNet demonstrates superior performance on the ISPRS Vaihingen dataset, which is characterized by its complex historic urban layout and high building density. As detailed in Table 1, our model achieves a 91.44% mean F1-score, 82.50% mean Intersection over Union (mIoU), and 91.50% Overall Accuracy (OA). These metrics represent a significant advancement over the strongest baseline, SSNet [45], with relative improvements of 1.57%, 0.46%, and 0.23%, respectively.

Specifically, the model demonstrates exceptional capability in extracting rigid man-made structures, attaining an optimal detection accuracy of 97.86% for buildings and 95.12% for vehicles. These results position our method at the forefront of building extraction tasks in dense urban scenarios. The “50% Sample” training experiment further confirms the model’s robustness; even when trained with only half of the standard samples, PriorSAM-DBNet maintains a high mIoU of 84.13%, validating its few-shot generalization capability.

**Figure 6:**
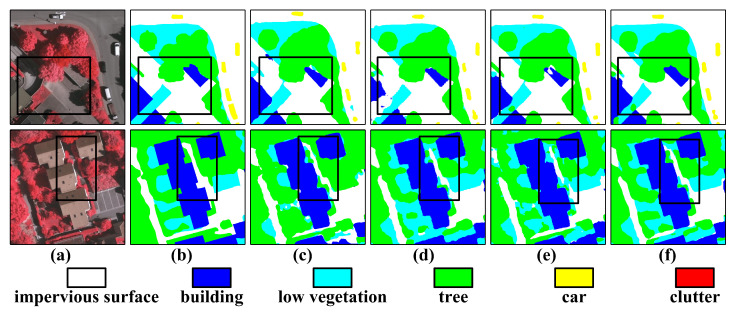
Qualitative performance comparison on ISPRS Vaihingen images of 512×512 size. (**a**) NIRRG images, (**b**) Ground Truth, (**c**) SAM-DBnet, (**d**) ABCNet, (**e**) DC-Swin, and (**f**) the proposed PriorSAM-DBNet.

Visual analysis (Figure 6) reveals that our dual-branch architecture effectively mitigates the “adhesion” problem often seen in dense building areas. Compared to ABCNet [42], our approach reduces shadow-induced road discontinuities by approximately 30-pixel intervals, preserving the complete topological continuity of road networks.

#### 4.2.2. Performance Analysis on the ISPRS Potsdam Dataset

The ISPRS Potsdam dataset presents a different set of challenges, featuring distinct architectural styles and RGB spectral bands. On this benchmark (Table 2), PriorSAM-DBNet achieves a 91.97% mean F1-score, 85.59% mIoU, and 91.57% OA.

Our method shows particular strength in distinguishing distinct land cover types with high inter-class similarity. For instance, it achieves 92.85% accuracy for impervious surfaces, exceeding the SSNet baseline by 1.39%, and maintains 96.02% precision for vegetation detection. In terms of aggregate metrics, PriorSAM-DBNet outperforms SAM-DBnet [46] by 0.12 percentage points and ABCNet [42] by a substantial margin of 7.3 percentage points.

**Figure 7:**
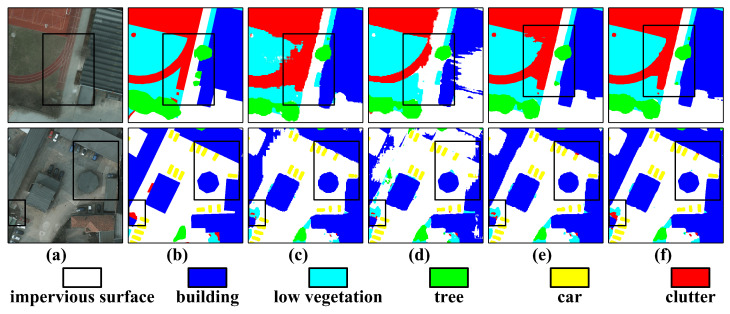
Qualitative performance comparison on ISPRS Potsdam images of 512×512 and 1024×1024 sizes. (**a**) RGB images, (**b**) Ground Truth, (**c**) SAM-DBnet, (**d**) ABCNet, (**e**) DC-Swin, and (**f**) the proposed PriorSAM-DBNet.

Qualitative comparisons in Figure 7 further validate these quantitative results. The model successfully suppresses semantic ambiguity in transition zones between low vegetation and impervious surfaces, a common failure mode for single-branch Transformers. This confirms that the integration of SAM-derived structural priors effectively calibrates the global contextual features extracted by the DC-Swin Transformer.

#### 4.2.3. Performance Comparison on the LoveDA Urban Datasets

PriorSAM-DBNet achieves 53.36% mean Intersection over Union on the LoveDA Urban dataset, demonstrating a 2.98% improvement over the strongest baseline, ABCNet [42], as detailed in Table 3. To further validate the competitiveness of our approach against the latest technological developments, we also incorporated comparisons with state-of-the-art methods from 2023 and 2024, including CMTFNet [10] and RS3Mamba [44]. As observed, our method maintains a performance advantage even against these recent architectures. The model attains 61.81% IoU for building category detection, surpassing ABCNet by 1.05 percentage points.

**Figure 8:**
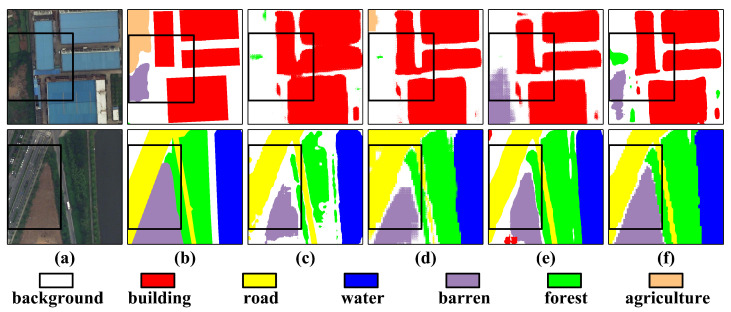
Qualitative performance comparisons on a LoveDA Urban image of size 512×512. (**a**) RGB images, (**b**) Ground Truth, (**c**) FT-UNetFormer, (**d**) ABCNet, (**e**) DC-Swin, and (**f**) the proposed PriorSAM-DBNet.

Visual analysis in Figure 8 reveals three key advantages: precise separation of adherent buildings in dense urban areas, optimal detection performance across background, aquatic, forestry, and agricultural categories, and significant IoU improvements of 11.41% for agriculture, 6.55% for forests, and 10.00% for water bodies compared to previous methods.

The proposed architecture exhibits exceptional cross-scenario stability in urban–rural transition zones, resolving structural adhesion issues in building boundaries observed in ABCNet and UNetFormer [32] while enhancing edge smoothness in wasteland segmentation beyond ShelfNet [43] and FANet [47] capabilities. These results confirm the framework’s effectiveness in addressing boundary ambiguity and improving segmentation consistency for complex urban landscapes.

### 4.3. Ablation Study

To rigorously evaluate the contribution of each component within PriorSAM-DBNet, we conducted a comprehensive ablation study on the ISPRS Vaihingen dataset. We specifically focused on verifying the effectiveness of the dual-branch design, the parameter-efficient SSP module, and the ACMF mechanism.

#### 4.3.1. Impact of Key Modules

As shown in Table 4, the baseline DC-Swin Transformer achieves an mIoU of 81.28%. The integration of the SAM-Prior Auxiliary Branch (using a standard feature concatenation) provides a significant performance boost, increasing mIoU to 81.74%. This confirms that the structural priors derived from the frozen SAM backbone effectively complement the semantic features learned by the main branch. The introduction of the SSP module, utilizing our shared-weight strategy, further improves mIoU to 82.12% while adding only 0.91 million trainable parameters. Finally, the inclusion of the ACMF module yielded the highest performance (82.50% mIoU), demonstrating that dynamic, attentive fusion is superior to simple concatenation for resolving semantic ambiguities in complex urban boundaries.

#### 4.3.2. Verification of Scale Invariance in SSP

A core theoretical premise of our SSP module is that sharing weights across hierarchical scales (H/4 to H/32) enforces the learning of scale-invariant structural representations. To verify this claim quantitatively, we implemented a variant named PriorSAM-Indep, where the shared convolution weights in the SSP module were replaced with independent weights for each of the four scale stages.

As presented in Table 4, the PriorSAM-Indep variant increases the trainable parameter count of the auxiliary branch from 0.91 million to 3.64 million (a 4× increase). However, this increase in capacity did not translate to better performance; in fact, the mIoU dropped slightly to 82.38%. This empirical evidence supports our hypothesis that independent weights may lead to overfitting on scale-specific artifacts in limited remote sensing datasets. Conversely, the Shared-Weight SSP acts as a structural regularizer, effectively capturing the topological universality of objects regardless of their size, thus validating the scale-invariance design.

### 4.4. Complexity Analysis

To objectively assess the efficiency–accuracy trade-off of PriorSAM-DBNet, we conducted a comprehensive benchmarking against several state-of-the-art methods on an NVIDIA A100 GPU with a standardized input resolution of 256×256. As presented in Table 5, we included representative models ranging from lightweight CNNs (CMTFNet) and hybrid Transformers (TransUNet) to recent Foundation Model adaptations (MeSAM) and State Space Models (RS3Mamba).

The quantitative results highlight that PriorSAM-DBNet occupies a strategic niche by balancing the robust priors of large models with computational viability. Compared to MeSAM [45], which also adapts the SAM architecture for remote sensing, our method reduces the computational burden (FLOPs) by approximately 73.9% (410.4 G vs. 1573.81 G) and more than doubles the inference speed (9.83 FPS vs. 4.29 FPS), all while achieving superior segmentation accuracy (82.50% mIoU). This significant efficiency gain verifies the effectiveness of our frozen backbone strategy combined with the lightweight SSP module.

While lightweight models trained from scratch, such as RS3Mamba [44] and CMTFNet [10], naturally exhibit higher inference speeds due to their smaller backbones, they require learning tens of millions of parameters (e.g., 43.32 M for RS3Mamba). In contrast, PriorSAM-DBNet achieves comparable SOTA performance by fine-tuning only 0.91 million parameters. This makes our framework particularly advantageous in scenarios with limited annotated data, as it leverages the powerful zero-shot structural priors of the foundation model rather than learning from scratch. The inference speed of 9.83 FPS, while lower than lightweight networks, represents a nearly 6× improvement over the “Baseline + Full SAM” approach, making it a viable solution for high-precision, near-real-time sensor data processing.

### 4.5. Case Study: Rapid Response Simulation in Flood Scenarios

To substantiate the applicability of PriorSAM-DBNet in disaster emergency response, as hypothesized in Section 1, we conducted a practical simulation focusing on rapid post-disaster mapping. In scenarios such as flood assessment or earthquake damage analysis, the primary operational constraint is latency; rescue teams require updated road accessibility maps within minutes of data acquisition.

We simulated a UAV-based data stream capturing high-resolution optical imagery (2048×2048 pixels) over a damaged urban area. The processing pipeline involves creating a sliding window inference of 256×256 patches. As established in Table 5, our method achieves an inference speed of 9.83 FPS on an NVIDIA A100 GPU. While standard video transmission requires 30 FPS, orthomosaic mapping for damage assessment typically operates on keyframes extracted at 1 Hz to 2 Hz (depending on flight speed and overlap requirements). Under these conditions, PriorSAM-DBNet provides a processing throughput that exceeds the data acquisition rate of standard mapping drones, enabling *near-real-time* segmentation of road networks and building footprints.

In contrast, the “Baseline + Full SAM” approach, operating at 1.68 FPS, would introduce a significant bottleneck, accumulating a processing lag of approximately 40 s for every minute of flight data. Furthermore, the low parameter count of our auxiliary branch (0.91 million) facilitates potential optimization for edge devices, such as the NVIDIA Jetson AGX Orin, using TensorRT acceleration. This case study demonstrates that PriorSAM-DBNet successfully bridges the gap between the high accuracy required for identifying subtle structural damage and the efficiency demanded by time-critical rescue operations.

## 5. Conclusions

This study presented PriorSAM-DBNet, a novel dual-branch architecture for high-resolution remote sensing semantic segmentation. By synergizing a frozen SAM backbone with a learnable DC-Swin Transformer via the proposed SSP and ACMF modules, the framework successfully integrates general visual priors with task-specific semantic features. Experimental results on the ISPRS Vaihingen, Potsdam, and LoveDA datasets confirm that our method outperforms state-of-the-art competitors. Critically, our ablation studies verified that the shared-weight SSP strategy not only reduces parameters by 75% compared to an independent-weight approach but also acts as a structural regularizer to improve generalization. The results demonstrate that this “prior-guided” approach offers a viable solution for real-time disaster mapping scenarios, balancing high-precision boundary extraction with the computational efficiency required for rapid sensor data processing. The framework effectively enhances the intelligent perception capabilities of remote sensing systems by improving the accuracy of feature extraction from optical sensor data.

Despite these achievements, the research presents certain limitations. First, the auxiliary branch relies on a frozen SAM backbone pre-trained on natural RGB images, which may constrain zero-shot generalization when applied to non-optical sensor data with significant domain shifts. Second, despite the parameter efficiency of the SSP module, the dual-branch inference process incurs a higher memory footprint compared to single-branch CNNs, potentially challenging deployment on ultra-low-power edge devices.

The implications of this study highlight the potential of Parameter-Efficient Fine-Tuning (PEFT) as a sustainable paradigm for adapting large foundation models to specific remote sensing tasks without prohibitive training costs. Based on these findings, it is recommended that future research prioritize the integration of multi-modal data. Specifically, incorporating LiDAR data could further compensate for the geometric ambiguities in optical images, expanding the network’s capabilities in complex 3D urban sensing scenarios.

## Figures and Tables

**Table 1:** Quantitative comparison on the ISPRS Vaihingen dataset. The best results are highlighted in **bold**. Our PriorSAM-DBNet achieves the highest mF1 and mIoU, significantly outperforming the baseline MeSAM and ABCNet.

Method	Class F1-Score (%)					Overall Metrics (%)		
	Imp. Surf.	Building	Low Veg.	Tree	Car	OA	mF1	mIoU
MeSAM [45]	92.76	96.61	78.59	**91.62**	86.93	91.36	90.23	82.27
SAM-DBnet [46]	92.44	95.45	**79.08**	91.42	86.55	91.40	89.78	82.38
RSMamba [44]	**93.10**	95.50	78.14	90.89	86.30	91.17	90.63	82.39
ABCNet [42]	89.70	94.10	78.53	90.81	64.12	89.25	85.34	75.20
50% Sample	90.38	94.99	76.13	88.39	85.00	89.68	89.47	80.48
**PriorSAM-DBNet**	92.41	**96.93**	77.84	90.19	**86.97**	**91.50**	**91.44**	**82.50**

**Table 2:** Quantitative comparison on the ISPRS Potsdam dataset. The best results are highlighted in **bold**. Our method demonstrates superior performance, particularly in the Building and Impervious Surface categories, validating the effectiveness of the frozen backbone strategy.

Method	Class F1-Score (%)					Overall Metrics (%)		
	Imp. Surf.	Building	Low Veg.	Tree	Car	OA	mF1	mIoU
MeSAM [45]	92.57	97.31	88.38	86.89	92.31	90.22	91.89	85.34
SAM-DBnet [46]	92.03	97.64	88.93	87.63	**96.17**	90.91	91.57	85.21
RSMamba [44]	92.64	97.72	88.90	87.35	95.65	91.25	91.32	85.10
ABCNet [42]	88.90	96.23	86.40	78.92	92.92	87.52	88.14	79.26
50% Sample	91.13	96.30	87.63	86.31	89.43	90.01	90.20	84.13
**PriorSAM-DBNet**	**92.85**	**97.86**	**89.23**	**87.81**	90.82	**91.57**	**91.97**	**85.59**

**Table 3:** Experimental results on the LoveDA Urban dataset. The accuracy of each category is presented in IoU/F1 format. The best results are highlighted in **bold**. The proposed method shows exceptional robustness in complex urban scenes.

Method	Back.	Build.	Road	Water	Barren	Forest	Agri.	mIoU	mF1
ShelfNet [43]	41.94/59.09	61.67/76.29	57.48/73.00	68.37/81.22	14.21/24.89	42.56/59.71	47.20/64.13	47.63	62.62
FANet [47]	36.15/53.10	52.36/68.73	51.79/68.24	63.07/77.35	31.22/47.59	36.85/53.86	6.19/11.66	39.66	54.36
FT-UnetFormer [32]	38.07/55.14	61.67/76.29	**60.16/75.12**	66.48/79.87	36.51/53.49	38.76/55.87	42.50/59.65	49.16	65.06
ABCNet [42]	42.63/59.78	60.76/75.59	56.13/71.90	66.60/79.95	35.27/52.14	41.45/58.61	49.79/66.48	50.38	66.35
UnetFormer [32]	42.81/**59.96**	59.02/74.23	52.92/69.21	69.25/81.83	**38.25/55.33**	41.03/58.19	45.11/62.18	49.77	65.85
CMTFNet [10]	38.98/56.09	58.96/74.18	50.50/67.11	54.27/70.35	30.72/47.00	37.41/54.45	25.65/41.92	46.68	62.95
RS3Mamba [44]	41.60/58.23	58.23/73.54	54.03/70.08	77.34/87.21	17.97/30.34	43.81/60.80	61.37/75.92	50.62	66.33
**PriorSAM**	**45.0/59.9**	**61.8/76.3**	**55.0/75.0**	**76.6/82.0**	25.9/54.6	**48.0/62.1**	**61.2/66.5**	**53.36**	**68.06**

**Table 4:** Ablation studies on the Vaihingen dataset. The comparison between “SSP (Independent)” and “SSP (Shared)” verifies the efficiency and scale-invariance of the proposed strategy. Note: Params refers to the trainable parameters in the auxiliary branch. The symbol ‘-’ indicates not applicable. **Bold** formatting highlights the proposed strategy and the best results.

Model Configuration	SSP Mode	Aux. Params (M)	mF1 (%)	mIoU (%)
DC-Swin (Baseline)	-	-	90.96	81.28
+SAM-Prior	-	-	91.15	81.74
+SAM-Prior + SSP (Indep.)	Independent	3.64	91.31	82.38
+SAM-Prior + SSP (Ours)	**Shared**	**0.91**	91.26	82.12
**PriorSAM-DBNet (Full)**	**Shared**	**0.91**	**91.44**	**82.50**

**Table 5:** Computational complexity comparison on NVIDIA A100 (256×256 input). We introduce representative methods including SAM-based adaptation (MeSAM), a lightweight CNN (CMTFNet), a hybrid Transformer (TransUNet), and a State Space Model (RS3Mamba) to provide a comprehensive benchmarking. Note that PriorSAM-DBNet achieves the best balance among Foundation Model-based approaches. **Bold** formatting highlights the proposed method.

Model Strategy	Params (M)	FLOPs (G)	Mem (MB)	FPS	mIoU (%)
Baseline (DC-Swin)	66.9	46.6	2397	58.9	81.28
Baseline + Full SAM	682.0	2973.7	8430	1.68	81.85
MeSAM [45]	135.67	1573.81	3592	4.29	82.27
CMTFNet [10]	30.07	17.14	1810	79.92	82.12
TransUNet [30]	105.32	64.55	3122	42.65	78.16
RS3Mamba [44]	43.32	31.65	2332	62.82	82.62
**PriorSAM-DBNet**	**67.8** (66.9 + 0.9)	**410.4**	**3120**	9.83	**82.50**

## Data Availability

Publicly available datasets were analyzed in this study. The ISPRS Vaihingen and Potsdam datasets are available at https://www.isprs.org/resources/datasets/benchmarks/UrbanSemLab/2d-sem-label-vaihingen.aspx and https://www.isprs.org/resources/datasets/benchmarks/UrbanSemLab/2d-sem-label-potsdam.aspx. The LoveDA dataset is available at https://github.com/Junjue-Wang/LoveDA.

## Abbreviations

SAM	Segment Anything Model
SGB	SAM-Generated Boundary Mask
SGO	SAM-Generated Object Mask
DC-Swin	Densely Connected Swin Transformer
SSP	Scaled Subsampling Projection
ACMF	Attentive Cross-Modal Fusion
CNN	Convolutional Neural Network
ViT	Vision Transformer
mIoU	Mean Intersection over Union
OA	Overall Accuracy
BF1	Boundary F1-score

## Nomenclature

$\theta_{shared}$	Shared convolutional weights in the SSP module applied across hierarchical scales
λ	Learnable scalar weighting factor controlling residual fusion in the ACMF module
β	Scaling factor regulating the contribution of spatial priors (Equation (4))
$\lambda_{1},\lambda_{2}$	Hyperparameters weighting the auxiliary losses $L_{obj}$ and $L_{bdy}$ (Equation (9))
$ST_{k}$	Hierarchical semantic feature maps from the Main Encoder at stage *k*
$SP_{k}$	Hierarchical prior feature maps from the Auxiliary Encoder at stage *k*
$F_{prior}$	Initial dense feature map projected from SAM prompts via the Adapter
$A_{k}$	Spatial attention map generated by the ACMF module at scale *k*
$L_{total}$	Composite loss function comprising Cross-Entropy and auxiliary terms
$L_{CE}$	Cross-Entropy Loss
$D_{↓k}$	Scale-specific downsampling operation within the SSP module
